# Methionine 274 Is Not the Determining Factor for Selective Inhibition of Histone Deacetylase 8 (HDAC8) by L-Shaped Inhibitors

**DOI:** 10.3390/ijms231911775

**Published:** 2022-10-04

**Authors:** Niklas Jänsch, Kim Leoni Lang, Franz-Josef Meyer-Almes

**Affiliations:** Department of Chemical Engineering and Biotechnology, University of Applied Sciences Darmstadt, Haardtring 100, 64295 Darmstadt, Germany

**Keywords:** HDAC inhibitors, binding selectivity, thermo stability, HDAC8 muteins

## Abstract

HDAC8 is an important target in several indication areas including childhood neuroblastoma. Several isozyme selective inhibitors of HDAC8 with L-shaped structures have been developed. A theoretical study has suggested that methionine 274 (M274) would act as a “switch” that controls a transient binding pocket, which is induced upon binding of L-shaped inhibitors. This hypothesis was experimentally examined in this study. The thermostability and functionality of HDAC8 wildtype and mutant variants with exchanged M274 were analyzed using biophysical methods. Furthermore, the binding kinetics of L-shaped and linear reference inhibitors of these HDAC8 variants were determined in order to elucidate the mode of interaction. Exchange of M274 has considerable impact on enzyme activity, but is not the decisive factor for selective recognition of HDAC8 by L-shaped inhibitors.

## 1. Introduction

**Figure 1:**
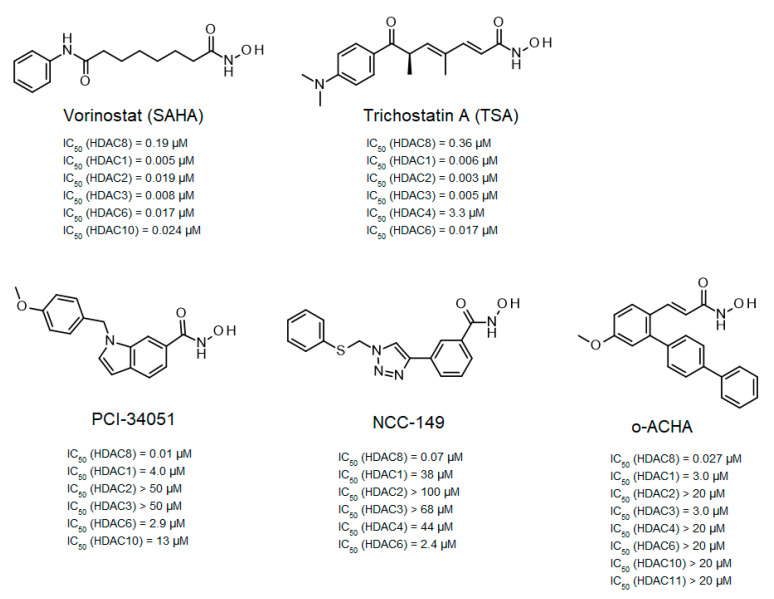
Structures of linear and L-shaped HDAC inhibitors used in this study. IC_50_-values of SAHA and PCI-34051 are from Balasubramanian et al. [12], NCC-149 from Suzuki et al. [13], and o-ACHA from Huang et al. [14].

Epigenetic regulation by acetylation and deacetylation of proteins is an important mechanism by which living cells control essential cellular processes. Initial observations have indicated that histones can be acetylated and thereby modulate chromatin structure and gene expression. This has stimulated comprehensive research focusing on the actors responsible for posttranslational modifications and especially the acetylation of histones. The acetylation state of histones and other proteins in cells is reversibleand depends on a dedicated balance of acetylation maintained by acetyltransferases and deacetylation by the action of histone deacetylases. Proteome analyses revealed that protein acetylation is not focused on histone class, but rather involves thousands of proteins in all cellular compartments, suggesting that acetylation is a common mechanism by which cellular processes are controlled, very similar to protein phosphorylation [1,2] Therefore, it is no surprise that deregulation of protein acetylation is associated with multiple diseases, including cancer [3,4,5] as probably the most important indication area, and also nephrological and neurodegenerative diseases [6,7] Human histone deacetylase 8 (HDAC8) is a zinc-dependent member of HDAC class I, which deacetylates numerous substrates in the nucleus and cytoplasm [8,9,10], and is a proven target for childhood neuroblastoma [11]. Selective inhibition of HDAC8 caused strong anti-neuroblastoma effects without toxicity in xenograft mouse models [11]. This observation stimulated the development of several selective HDAC8 inhibitors (Figure 1).

Among the first inhibitors found to have extraordinary selectivity for HDAC8 were PCI-34051 and NCC-149 [12,13]. The hydroxamic acid PCI-34051 has an IC_50_-value of 10 nM and a selectivity more than 200-fold higher than other HDAC isozymes. Consequently, PCI-34051 has been the most commonly used reference compound for studying the biological role of HDAC8 in cellular systems [11,15,16,17]. Huang et al. developed branched ortho-aryl-N-hydroxycinnamides as HDAC8-selective inhibitors, including o-ACHA which inhibits HDAC8 with an activity similar to PCI-34051 and has antiproliferative effects against several cancer cell lines [14]. In contrast, most linear hydroxamic acid HDAC inhibitors, including clinically approved suberoylanilide hydroxamic acid (SAHA, vorinostat) and trichostatin A (TSA), are unspecific pan-inhibitors effecting more or less all zinc-dependent HDACs. It appears that many HDAC8-selective inhibitors have L-shaped forms [12,13,18,19]. This particular aspect was investigated in a comprehensive study by Marek et al., who showed that HDAC8-selective inhibitors adopt an L-shaped conformation when binding to a HDAC8-specific pocket between Y306 and the L1 and L6 loops [20]. Crystal structures of complexes between selective inhibitors and HDAC8 from *Schistosoma mansoni* (smHDAC8) showed preferential molecular interactions with the catalytic tyrosine (corresponding to Y306 in human HDAC8) and the L6 loop, which is more flexible than the L1 loop. Although HDAC8 from *Schistosoma mansoni* (smHDAC8) shares only 41% sequence identity with human HDAC8, both structures show a very high degree of overlap, and the active site differs only by one amino acid in the L6 loop, where H292 in smHDAC8 corresponds to M274 in human HDAC8. L-shaped inhibitors show very similar binding poses in wildtype smHDAC8 and “humanized” mutant variant smHDAC8_H292M_ [20]. In other HDAC isozymes, a L1–L6 lock sterically prevents L-shaped inhibitor binding, which might explain why L-shaped inhibitors preferentially bind to HDAC8. In a computational study using flexible docking and molecular dynamics simulations, Yao et al. suggested that the flexibility of methionine 274 (M274) and the L-shaped structure of inhibitors might be the most important determinants for selective inhibition of HDAC8 [21]. Binding of L-shaped inhibitors was proposed to induce a conformational change of M274 from flipped-out to flipped-in conformation, which should open the HDAC8-selective pocket. In other HDAC isozymes, methionine is replaced by leucine, which lacks the flexibility of M274 in molecular dynamics simulations. Consequently, L-shaped inhibitors were assumed to be unable to induce opening of the selectivity pocket between the L1 and L6 loops [21]. Since selective L-shaped inhibitors bind in very similar poses to wildtype smHDAC8 and smHDAC8_H292M_ in crystal structures, where the critical H292 is exchanged with methionine [20], it appears disputable whether the postulated function of this amino acid as a pivotal switch to control the exposure of the selective sub-pocket would be the determining mechanism for selective HDAC8 inhibition. To clarify the role of M274 in isozyme-selective HDAC8 inhibition, we performed a comprehensive series of experiments to investigate the functionality, thermostability, and ligand binding of human HDAC8 wildtype and mutant variants. herein the experiments, M274 was exchanged by either leucine, which is conserved among most other human HDAC isozymes, or alanine, which is thought to impose less steric restrictions and, therefore, should resemble a permanently open and well accessible HDAC8-selective sub-pocket.

## 2. Results and Discussion

More than 80 crystal structures of HDAC8 (54 human and 30 from *Schistosoma mansoni*) have been obtained in complex with a variety of inhibitors. However, no diffractable crystals have been obtained from the apo-enzyme, which has been attributed to the extraordinary flexibility of HDAC8 [22]. Ligand binding and enzyme activity of HDAC8 have been shown to depend largely on the flexibility of the neighboring loops [23,24,25,26,27,28]. The high malleability of the binding region allows the accommodation of ligands that are highly chemically and structurally divergent, including SAHA [25], PCI-34051 [20], tropolones [29], largazole analogs [26], 1,3-benzo-thiazine-2-thiones [17], meta-substituted benzhydroxamic acids [18], and so-called linkerless HDAC8 inhibitors [19]. Crystal structures of HDAC8 complexes with non-selective linear inhibitors showed no participitation of M274 during side pocket formation [25]. By analyzing crystal structures of L-shaped inhibitors bound to smHDAC8 from *Schistosoma mansoni*, a special selective binding pocket was observed between the catalytic tyrosine and the L1–L6 loop [20]. A computational study suggested that this selective binding pocket is a transient binding pocket, which opens upon binding of L-shaped inhibitors, and is controlled by movement of M274 [21]. Moreover, this structural change was not observed when M274 was replaced by leucine in the same computational study, leading to the hypothesis that M274 is the key factor responsible for selective HDAC8 inhibition [21].

**Figure 2:**
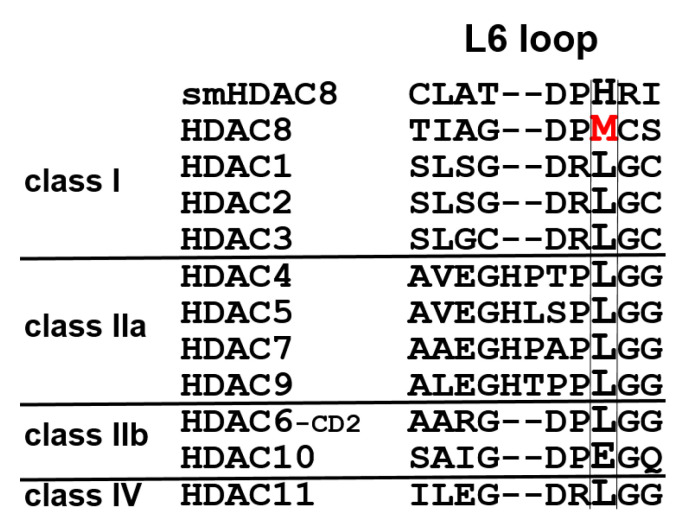
Multiple sequence alignment of the L6 loop across all human zinc-dependent HDAC isozymes and smHDAC8 from *Schistosoma mansoni*. M274 of human HDAC8 is highlighted in bold and red.

**Figure 3:**
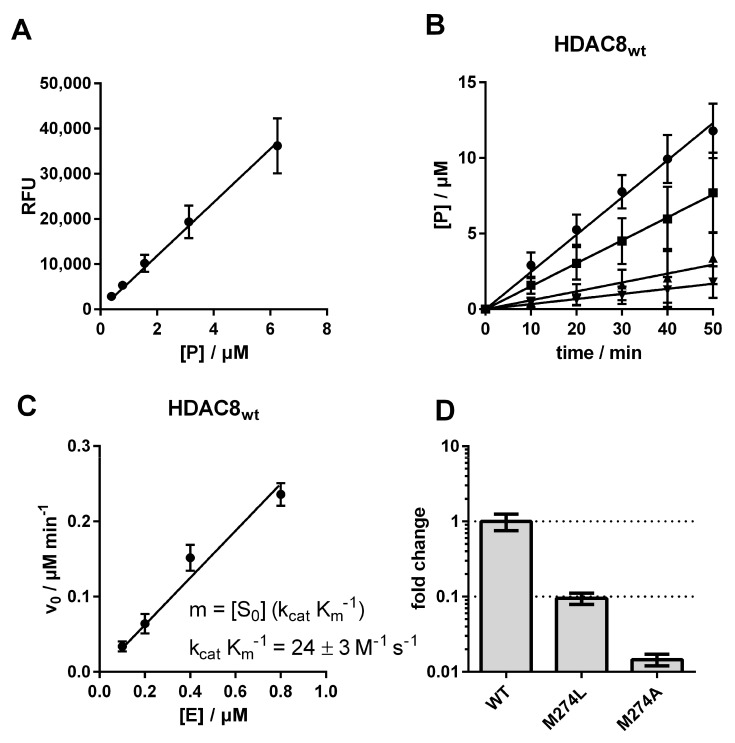
Determination of catalytic efficiencies for HDAC8. (**A**) Product calibration using free AMC. (**B**) Progress curves for the conversion of 200 µM of the artificial substrate Boc-Lys(Ac)-AMC by ● 0.8 µM, ∎ 0.4 µM, ▲ 0.2 µM, and ▼ 0.1 µM HDAC8_wt_. (**C**) Plot of initial velocity against enzyme concentration. (**D**) Comparison of the catalytic efficiencies. Data show mean values and standard deviations, *n* ≥ 3.

To address this important research question, mutant variants of human HDAC8 were generated using site-directed mutagenesis, where M274 was exchanged either against leucine, which is conserved in all other human zinc-dependent HDACs with exception of HDAC10 (Figure 2), or against alanine, which was chosen because of its small side chain. We measured the impact of these two mutations on the substrate conversion of the artificial substrate Boc-Lys(Ac)-AMC, using the assay developed by Werbeck et al. (Figure 3 and Figure S1) [30,31]. 

**Figure 4:**
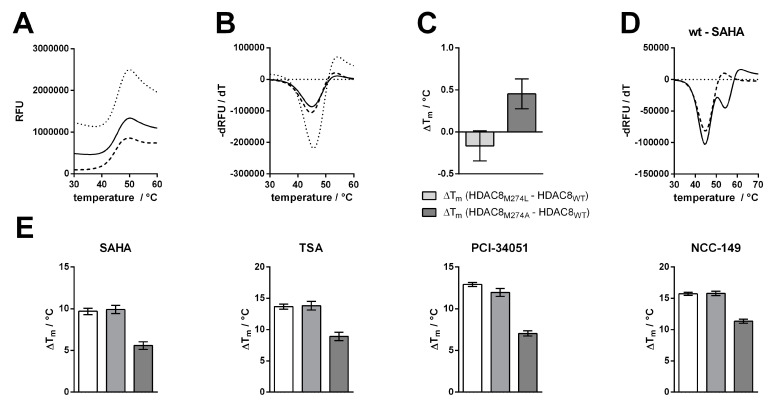
Determination of protein melting points and thermal shift assay. (**A**) Raw fluorescence data for the thermal denaturation of HDAC8_wt_ (meshed line), HDAC8_M274L_ (solid line), and HDAC8_M274A_ (dotted line). (**B**) First derivative for the thermal denaturation of HDAC8_wt_ (meshed line), HDAC8_M274L_ (solid line), and HDAC8_M274A_ (dotted line). (**C**) Comparison of T_m_ values between the mutant variants and the wild type. (**D**) Thermal shift assay showing unbound HDAC8_wt_ (meshed line) and SAHA-bound HDAC8_wt_ (solid line). (**E**) Thermal shifts for SAHA, TSA, PCI-34051, and NCC-149 for HDAC8_wt_ (white bar), HDAC8_M274L_ (light gray bar), and HDAC8_M274A_ (dark gray bar). For the thermal shift assay, 12.5 µM HDAC8 and 125 µM of indicated inhibitor were used. Data show mean values and standard deviations, *n* ≥ 3.

The catalytic efficiency of the human wildtype HDAC8 (HDAC8_wt_) was determined to be 24 ± 3 M^−1^ s^−1^, in agreement with the value measured by Kunze et al. under comparable conditions (38 ± 4 M^−1^ s^−1^) [31]. By exchanging M274 to leucine, the catalytic efficiency decreased about 10-fold to a value of 2.3 ± 0.1 M^−1^ s^−1^, and an exchange to alanine yielded an even higher drop of catalytic efficiency down to a value of 0.4 ± 0.01 M^−1^ s^−1^, which was only about 1% compared with HDAC8_wt_ activity (Figure 3D, Table S1). To check whether this dramatic change in enzyme activity was caused by changes to the structural integrity and stability of the enzyme, we analyzed thermal protein denaturation using differential scanning fluorimetry (Figure 4). There was no significant difference in protein stability between HDAC8_wt_ and HDAC8_M274L_, and only slightly less stability of HDAC8_M274A_ compared with HDAC8_wt_, indicating that the overall structural integrity of HDAC8_wt_ was essentially unaffected by M274L and M274A exchanges (Figure 4A–C).

The dramatic differences in catalytic efficiencies reflect altered substrate recognition and turnover numbers, and might also have been caused by subtle structural changes of the active site. More specifically, M274 appears to be important for the recognition and turnover of the artificial substrate Boc-Lys(Ac)-AMC, and is probably an important determinant for the spectrum of HDAC8-specific substrates in vivo.

**Figure 5:**
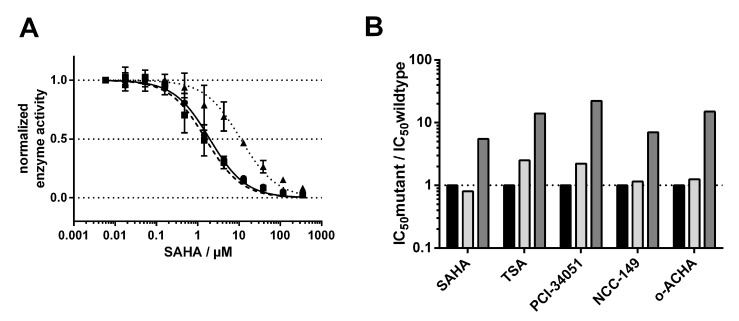
IC_50_ determination and comparison. (**A**) Dose–response curve for the IC_50_ determination of SAHA for HDAC8_wt_ (●, solid line), HDAC8_M274L_ (∎, meshed line), and HDAC8_M274A_ (▲, dotted line). (**B**) Comparison of IC_50_ values for different linear and L-shaped inhibitors for HDAC8_wt_ (black bar), HDAC8_M274L_ (light gray bar), and HDAC8_M274A_ (dark gray bar).

Next, we investigated how the exchange of M274 to leucine and alanine impacted the binding behavior of a selection of commonly used and well-studied linear inhibitors (SAHA, TSA), as well as L-shaped inhibitors (PCI-34051, NCC-149, and o-ACHA), which are known to be HDAC8-selective [20]. We measured IC_50_ values of selected inhibitors against HDAC8_wt_ and mutant variants HDAC8_M274L_ and HDAC8_M274A_ (Table S2). Interestingly, linear and L-shaped inhibitors showed very similar inhibition patterns against all HDAC8 variants (Figure 5B). To our surprise, the IC_50_ values between HDAC8_wt_ and HDAC8_M274L_ showed no significant differences (Figure 5 and Figure S2).

This observation contradicted the postulation of a computational study which suggested that leucine instead of methionine is unable to act as a switch that opens a side pocket for the binding of HDAC8-selective L-shaped inhibitors [21]. We anticipated that the replacement of methionine by the smaller amino acid alanine in HDAC8_M274A_ would create a constitutively open HDAC8-selective pocket between the L1- and L6-loop, with facilitated access for L-shaped inhibitors. Contrary to our expectation, IC_50_-values of all inhibitors, no matter whether linear or L-shaped, were about 10-fold higher than for HDAC8_wt_ or HDAC8_M274L_ (Figure 5B and Figure S2), suggesting enforced hydrophobic interactions between inhibitors and M274. These hydrophobic interactions might also be formed when M274 is replaced by leucine with a similar hydrophobic side chain, but not by the much smaller alanine at this position. These findings clearly show that M274 is involved in general inhibitor binding, but disprove a pivotal role for M274 as a “switch” for the selective inhibition of HDAC8 by L-shaped inhibitors.

To support the observations made by IC_50_ measurements, we tested for thermal stabilization of all HDAC8 variants in the presence of linear and L-shaped inhibitors (Figure 5). Thermal shifts were calculated by the difference between the first and second melting events. We assumed that the second melting event involved the ligand-stabilized protein, because this event only occurs in the presence of a ligand (Figure 4D and Figure S3). The first melting event was mainly unaffected by ligand addition, and resulted in the value for the protein without ligand. In agreement with the IC_50_ results, we saw no differences in the thermal shifts of HDAC8_wt_ or HDAC8_M274L_ in the presence of inhibitors, whether L-shaped or linear (Figure 4E and Figure S2). Furthermore, we confirmed the increase in IC_50_ for the HDAC8_M274A_ variant, which showed smaller thermal shifts. HDAC8_M274A_ is about 4–6 °C less stabilized by ligand addition than either the wild type or the leucine mutant variant. These results support the enzyme activity data that was obtained, and demonstrate that the interaction between HDAC8_M274A_ and inhibitors was weakened with respect to HDAC8_wt_.

Taken together, the data show that M274 is not the sole determining factor for selective binding of L-shaped inhibitors to HDAC8. In particular, M274 does not control the exposure of the HDAC8-selective pocket.

**Figure 6:**
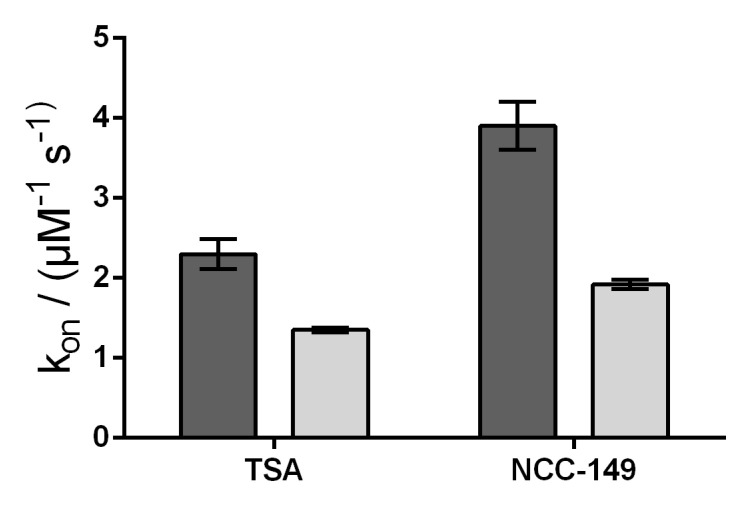
Comparison of the rate constants of association for TSA and NCC-149 between HDAC8_wt_ (dark gray bars) and HDAC8_M274A_ (light gray bars). Rate constants were determined by stopped-flow kinetics.

We further investigated the accessibility of the binding pocket by using stopped-flow experiments, and measured the rate constants for the binding of representative linear (TSA) and L-shaped (NCC-149) inhibitors to HDAC8_wt_ and HDAC8_M274A_. Because the active site of HDAC8 is flanked by three tryptophane residues, it is possible to measure a decrease in intrinsic fluorescence upon binding of a ligand to the active site. In theory, and under the assumption that M274 acts a gatekeeper for a transient binding pocket, the rate of the interaction depends on the movement of M274. Therefore, the interaction must be slower for HDAC8_wt_ than for the HDAC8_M274A_ mutant variant in which the postulated transient binding pocket is supposed constitutively open. Similar to the enzyme activity and protein stabilization experiments, there were no qualitative differences between the binding kinetics of linear and L-shaped inhibitors to HDAC8_wt_ and HDAC8_M274A_. However, we observed faster association rates for the binding of the inhibitors TSA and NCC-149 to HDAC8_wt_, compared with binding to HDAC8_M274A_, which was in contrast to our expectations noted above (Figure 6 and Figure S4). Thus, the association rate seems to reflect the lower affinity of all investigated inhibitors to HDAC8_M274A_ compared with HDAC8_wt_, and does not support the suggestion of M274 barring the access of L-shaped inhibitors to the HDAC8-selective sub-pocket.

### Flexibility around Binding Pocket of HDAC8

The extraordinary flexibility of loops around the active site is a specific feature of HDAC8. Different ligands induce alternative conformations of the L1 and L2 loop flanking the active site [25,26]. The active site binding pocket in an HDAC8-inhibitor complex can change from a narrow channel (PDB-ID 1T69), to a sub-open conformation with a second transient pocket (PDB-ID 1T64), to a structure with a wide open funnel-shaped pocket (PDB-ID 1VKG). Along with the L1 and L2 loop, the flipping of the F152 sidechain is one of the main characteristics in the dynamic interconversion between HDAC8 conformations [25,32]. Recently deposited crystal structures of HDAC8–ligand complexes show that L-shaped inhibitors induce a pronounced flip of the F152 side chain, and M274 also appears to be flexible and to interact with a ligand in some cases (e.g. PDB-IDs 6ODA, 6ODB, 6ODC).

**Figure 7:**
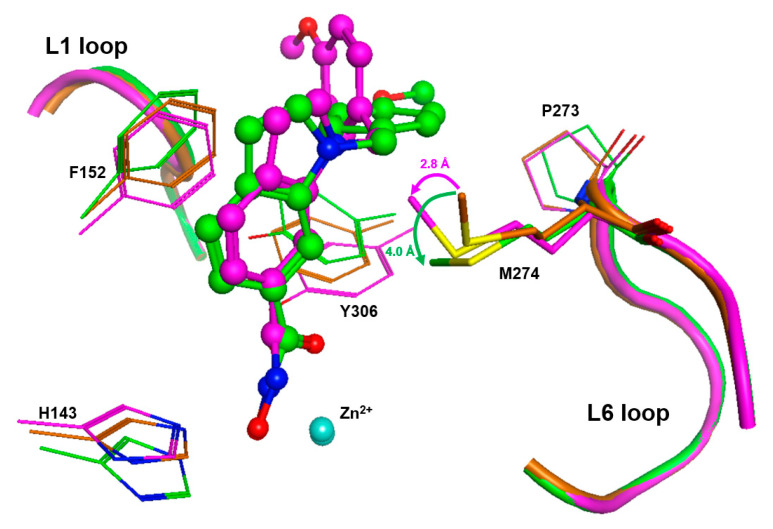
Close-up view of the binding pocket of human HDAC8. The unchanged conformation of HDAC8 (PDB-ID: 1T67, brown) is superimposed onto energy-minimized complex structures between HDAC8 (PDB-ID: 1T67) and PCI-34051 ligands (ball and sticks), with starting poses taken from tetrameric PDB-ID: 6HSF, chain A (green) and chain C (magenta). L1 and L6 loops are shown as tubes with the same color code. The catalytic zinc ions are displayed as cyan spheres. After some arrangement of the side chains of neighboring amino acids, PCI-34051 fits well into the selective HDAC8 pocket between Y306 and L1–L6 loops. Interestingly, the methyl group of M274 is not pushed away by the ligands, but is rather attracted, coming into proximity with the hydrophobic groups of the ligand.

To gain more insight in the possible role of M274 as a molecular switch controlling the opening of the transient HDAC8 sub-pocket between L1 and L6 loops, 11 representative crystal structures of human HDAC8 and the “humanized” HDAC8 from *Schistosoma mansoni* were analyzed to assess the structural conservation of amino acids flanking the active site pocket, including P273 and M274 from the L6 loop. This analysis revealed a low average RMSD value of 0.389 Å over 10 conserved amino acids flanking the active site pocket (Figures S5 and S6), indicating that the positions of these amino acids are highly defined. However, the analysis also indicates some flexibility around the binding pocket of HDAC8, in agreement with previous studies [26,28]. As expected, a closer look at distinct amino acids revealed that the positions of catalytic and, particularly, zinc-chelating amino acids remained almost unchanged, whereas those flanking the hydrophobic binding tunnel and the L6 loop showed more flexibility (Table S3). This is in agreement with numerous observations about the high malleability of the binding site in HDAC8 [26,27]. M274 in the L6 loop demonstrates structural deviations similar to those of F152 in the canonical binding tunnel (Table S3). In similar crystal structures of HDAC8 from *Schistosoma mansoni* (smHDAC8), L-shaped inhibitors have been found to bind into the HDAC8-selective sub-pocket between the L1–L6 loop and the catalytic tyrosine. The hydroxamate group of PCI-34051 chelates the catalytic zinc ion, and the methoxyphenyl head group forms Pi–Pi interactions with Y341 (Y306 in human HDAC8) and also H292. Furthermore, the methoxy group forms non-polar contacts with the sidechain of P291 in the L6 loop [20]. If H292 is exchanged by methionine corresponding to M274 in human HDAC8, the binding mode of PCI-34051 shows only minor changes, particularly regarding contact with the L6 loop. However, the ligand retains non-polar contact with P291 and also with the sidechain of M292 (alias M274) [20]. The crystal structure of smHDAC8_H292M_ (PDB-ID: 6HSF) is tetrameric. Interestingly, a superimposition of the four monomers gives another impressive argument for the intrinsic flexibility and adaptability to ligands of the HDAC8-selective pocket (Figure S5). The PCI-34051 ligand and the methyl group of M292 show different orientations in different monomers. The distances between the methyl carbon atoms of M292 among the four monomers vary between 0.24 and 2.2 Å. Notably, the methyl group of M292 in chain A of this crystal structure points to the closer oriented ligand, while the methyl group of M292 in chain C is moved away from the more distant ligand, demonstrating that the ligand does not necessarily push the methyl group aside (Figure S5B). To rationalize this observation, we inserted PCI-34051 ligands from chains A and C in the crystal structure of smHDAC8_H292M_ (PDB-ID: 6HSF) into the 3D structure of human HDAC8 (PDB-ID: 1T67), and performed subsequent energy minimization to optimize the binding poses within the selective HDAC8 binding pocket between L1–L6 loops and Y306. Notably, the methyl-group in the sidechain of M274 was not pushed away by the ligand but seemed to be attracted, coming into proximity with the ligand (Figure 7).

The orientation of the methyl group towards the ligand facilitates non-covalent hydrophobic interactions, which have been observed in crystal structures of smHDAC8_H292M_ in complex with PCI-34051 [20]. These results support our experimental data, which are in agreement with the suggestion of attracting hydrophobic interactions between M274 and active site binding inhibitors.

## 3. Materials and Methods

### 3.1. Recombinant Protein Production, Purification, and Mutant Variant Generation

pET14b vector (Novagen, EMD Millipore, Burlington, MA, USA) containing codon-optimized human HDAC8, fused to a n-terminal His6-SUMO tag, was used to express HDAC8 in *E. coli* BL21 (DE3) (New England Biolabs, Ipswich, MA, USA). Cells were grown in autoinduction media at 30 °C overnight, then harvested by centrifugation for 10 min at 8000× *g* and 4 °C. Cells were resuspended in lysis buffer (pH 8.0, 150 mM KCl, 50 mM Tris, 5 mM imidazole, 5 mM DTT, and 5 μg/mL DNAseI) and lysed by sonication. Lysates were clarified by centrifugation at 18,000× *g* and 4 °C for 30 min, then passed through a 0.45 μm PVDF syringe filter unit. Chromatography was conducted on a ÄKTA pure FPLC system (GE Healthcare). A 5 mL prepacked cOmplete His tag purification column was equilibrated with 5 cv of IMAC buffer A (pH 8.0, 150 mM KCl, 50 mM Tris, and 5 mM imidazole), then the lysate was pumped over the column and washed with 10 cv IMAC buffer A. Protein was eluted using a step gradient with IMAC buffer B (pH 8.0, 150 mM KCl, 50 mM Tris, 75 mM imidazole). Fractions containingHis6-SUMO-HDAC8 were pooled and 10 μg/mL His6 tagged SUMO protease was added whilst dialyzing against AIC buffer A (pH 7.0, 25 mM Tris, 50 mM NaCl) at 4 °C overnight. The dialysate was conducted to a second IMAC to remove His6-SUMO tag and His6-SUMO protease. Flow-through that contained HDAC8 was concentrated and further purified using a strong anion exchange column (MiniChrom Toyopearl GigaCap Q-650M, 5 mL, Tosoh Bioscience GmbH) and eluted with a linear gradient using AIC buffer B (pH 7.0, 25 mM Tris, 1 M NaCl). Fractions containing HDAC8 were pooled, concentrated to 1.5 mL, and 5 mM DTT was added to prevent oxidation. The final purification step included size exclusion chromatography with a HiLoad 16/600 Superdex 75 pg column (GE Healthcare, Chicago, IL, USA) equilibrated with SEC buffer (pH 8.0, 150 mM KCl, 50 mM Tris, 5% glycerol, 1 mM TCEP). The fractions containing HDAC8 were collected, concentrated, adjusted to 20 mg/mL, flash frozen with liquid nitrogen, and stored at −80 °C. Typical yields using this protocol were about 0.4% (mass wet cell pellet/mass protein) for the wildtype HDAC8. Primers used for point mutations are listed in Table S4.

### 3.2. Michaelis-Menten Parameters

Catalytic efficiencies were determined following the protocol by Werbeck et al. [30]. The indicated concentration of HDAC8 was mixed with 200 µM Boc-Lys(Ac)-AMC in MAL buffer (pH 8.0, 50 mM Tris, 137 mM NaCl, 2.7 mM KCl, 1 mM MgCl_2_, 1 mg/mL BSA) at 20 °C. Immediately after mixing the enzyme with substrate, 50 µL of the reaction mixture was removed and added to 50 µL developer solution (500 µM SAHA, 5 mg/mL trypsin in MAL buffer) for the measurement of a blank. Then, 50 µL aliquots were removed at 10, 20, 30, 40, 50 min and added to 50 µL developer solution. After the final timepoint, the measuring signal was developed for 15 min at 30 °C. Measurements were performed using a Pherastar fluorescence plate reader with a coumarin filter module. RFU for each timepoint were subtracted from RFU at 0 min and product concentration was calculated using external calibration of free AMC. Product concentration in µM was plotted against time in min, and the slope was calculated in GraphPad Prism using linear regression starting at 0/0 to yield initial velocity v0. Then v0 was plotted against enzyme concentration and slope was again forced to go through 0/0, and was divided by initial substrate concentration to yield catalytic efficiency k_cat_/K_m_. 

### 3.3. Protein Melting Points and Ligand Induced Thermal Stabilization

Protein melting curves were generated using the Quant Studio 5 real-time PCR system (Thermo Fisher Scientific, Waltham, MA, USA) and SYPRO orange dye. For that purpose, 0.5 mg/mL HDAC8 (12.5 µM) was mixed with a 10-fold concentration of SYPRO orange and 125 µM of the indicated inhibitor in SEC buffer, then preincubated for 1 h at 30 °C. Samples were heated using a linear gradient of 0.015 °C/s. Plotted curves represent the mean of three independent measurements.

### 3.4. IC_50_ Determination

Enzyme activity assay was executed in assay buffer (25 mM Tris-HCL pH 8.0, 50 mM NaCl, and 0.001 % (*v*/*v*) pluoronic F-68) in half area 96-well black microplates (Greiner Bio-One, Solingen, Germany). For IC_50_ determination, 10 nM HDAC8 was preincubated for 1 h with a serial dilution of the indicated compounds. The enzyme reaction was initiated by the addition of 20 µM Boc-Lys(TFA)-AMC (Bachem, Bubendorf, Switzerland). After substrate conversion at 30 °C for 1 h, the reaction was stopped by adding 1.67 µM suberoylanilide trifluoromethylketone (SATFMK). The deacetylated substrate was cleaved with 0.42 mg/mL trypsin to release fluorescent 7-amino-4-methylcoumarin (AMC, Amsterdam, The Netherlands), which was detected with a microplate reader (PHERAstar FS or BMG LABTECH) with fluorescence excitation at 360 nm and emission at 460 nm. IC_50_ values were calculated by generating dose-response curves in GraphPad Prism, and fitting those to a 4-parameter fit model.

### 3.5. Stopped-Flow Kinetics

Binding kinetics were measured using a BioLogic SFM3000 Stopped-Flow instrument, by mixing 0.5 µM HDAC8 with the indicated inhibitor concentrations in SEC buffer. Stopped-Flow was operated in fluorescence mode, and time-dependent change in tryptophane fluorescence was monitored over the course of 1 s with a data interval of 500 ns. Excitation wavelength was set to 281 nm with a 320 nm high pass cut-off filter. The output filter was set to 1 ms and voltage to 600. Kinetics were measured at 25 °C. Binding of inhibitors decreases the tryptophane fluorescence of HDAC8_wt_ and mutant variants.

### 3.6. Flexibility Analysis 

Eleven crystal structures of human HDAC8 in complex with different ligands (PDB-IDs 1T64, 1T67, 1T69, 1VGK, 1W22, 2V5W, 2V5X, 3F0R, 3F07, 3SFF, 5DC6), along with the crystal structure of smHDAC8_H292M_ from *Schistosoma mansoni* (PDB-ID 6HSF), were loaded into MOE 2020 software (Chemical Computing Group ULC, Montreal, QC, Canada) and superimposed after sequence alignment. Alignment and RMSD calculations were based on 10 conserved active site amino acids: H142, H143, F152, D178, H180, F208, D267, P273, M274 and Y306 (numbering according to human HDAC8). MOE 2020 was also employed to create the heatmap of pairwise RMSD values between these amino acids for all PDB entries. Where necessary, binding poses were energy minimized within a radius of 10 Å around the ligand, using MOE 2020 and applying an AMBER 14 forcefield.

## 4. Conclusions

The development of isozyme-selective HDAC inhibitors is challenging because of the sequentially and structurally highly conserved active site. Recently, a HDAC8-selective side pocket between the L1 and L6 loop was identified, which is required for the binding of L-shaped HDAC8-selective inhibitors [20]. M274 is located in the L6 loop and is unique to HDAC8, since all other human zinc-dependent HDACs except HDAC10 have a leucine at this position (Figure 2). Moreover, a computational study postulated that M274 acts as a switch to open the HDAC8-selective side pocket as an essential mechanism for selective HDAC8 inhibition [21].

In this experimental study we investigated the role of M274 for substrate and inhibitor recognition, using several HDAC8 mutant variants and a combination of biochemical and biophysical methods. Replacing M274 by leucine or alanine led to a dramatic drop in catalytic efficiency, demonstrating the important role of this amino in substrate recognition and turnover. However, HDAC8_wt_ and HDAC8_M274L_, which represent the active site of most other zinc-dependent HDACs, showed very similar inhibitor binding behavior for unselective linear and selective L-shaped inhibitors. Therefore, M274 is not the single and pivotal determinant for selective HDAC8 inhibition. Binding of all investigated inhibitors to HDAC8_M274A_ with a constitutively open HDAC8-selective pocket was less potent and slower compared with binding to HDAC8_wt_. This finding suggests an attracting hydrophobic interaction between M274 and active site binding inhibitors, which cannot be formed with the much smaller sidechain of alanine at this position.

In summary, M274 is important for catalytic efficiency, and its hydrophobic side chain can interact with active site binders. However, M274 does not serve as the decisive factor to control the opening of a transient HDAC8-selective pocket enabling preferential binding of L-shaped inhibitors to HDAC8.

## Data Availability

All supporting information is provided in the submitted Supplementary Materials.

## Supplementary Materials

The following supporting information can be downloaded at: https://www.mdpi.com/article/10.3390/ijms231911775/s1.
