# Supplementary material for: Methionine 274 Is Not the Determining Factor for Selective Inhibition of Histone Deacetylase 8 (HDAC8) by L-Shaped Inhibitors
Source: Int J Mol Sci. 2022 Oct 4;23(19):11775. doi: 10.3390/ijms231911775 (PMC9569839; doi:10.3390/ijms231911775)
Supplement: Supplementary file 1 [file ijms-23-11775-s001.zip › ijms-1940987-supplementary.pdf]

Supporting information:

Methionine 274 is not the determining factor for selective inhibition of histone deacetylase 8 (HDAC8) by L-shaped inhibitors

Niklas Jänsch[1], Kim Leoni Lang[1], Franz-Josef Meyer-Almes[1,*]

[1] Department of Chemical Engineering and Biotechnology, University of Applied Sciences Darmstadt, Haardtring 100, 64295 Darmstadt, Germany.

Content

Fig. S1: Determination of catalytic efficiencies for HDAC8.

Fig. S2: Dose-response curves for IC50 determination.

Fig. S3: Overview of thermal shift assay for the binding of SAHA, TSA, PCI-34051 and NCC-149 on $HDAC8_{wt}$, $HDAC8_{M274A}$, and $HDAC_{M274L}$.

Fig. S4: Determination of the rate constant of assotiation via stopped-flow.

Fig. S5: Overlay of HDAC8-monomers from the same crystal structure of tetrameric HDAC8 from *Schistosoma mansoni* (PDB-ID: 6HSF).

Fig. S6: RMSD-analysis of active site binding pocked based on superimposed crystal structures of HDAC8.

Table S1: Catalytic efficiencies and melting points for $HDAC8_{wt}$, $HDAC8_{M274L}$ and $HDAC8_{M274L}$.

Table S2: IC50, thermal shift and stopped-flow values for $HDAC8_{wt}$, $HDAC8_{M274L}$ and $HDAC8_{M274L}$.

Table S3: Pairwise RMSD-values smHDAC8 and human HDAC8 structures.

Table S4: Primers used for point mutations of HDAC8.

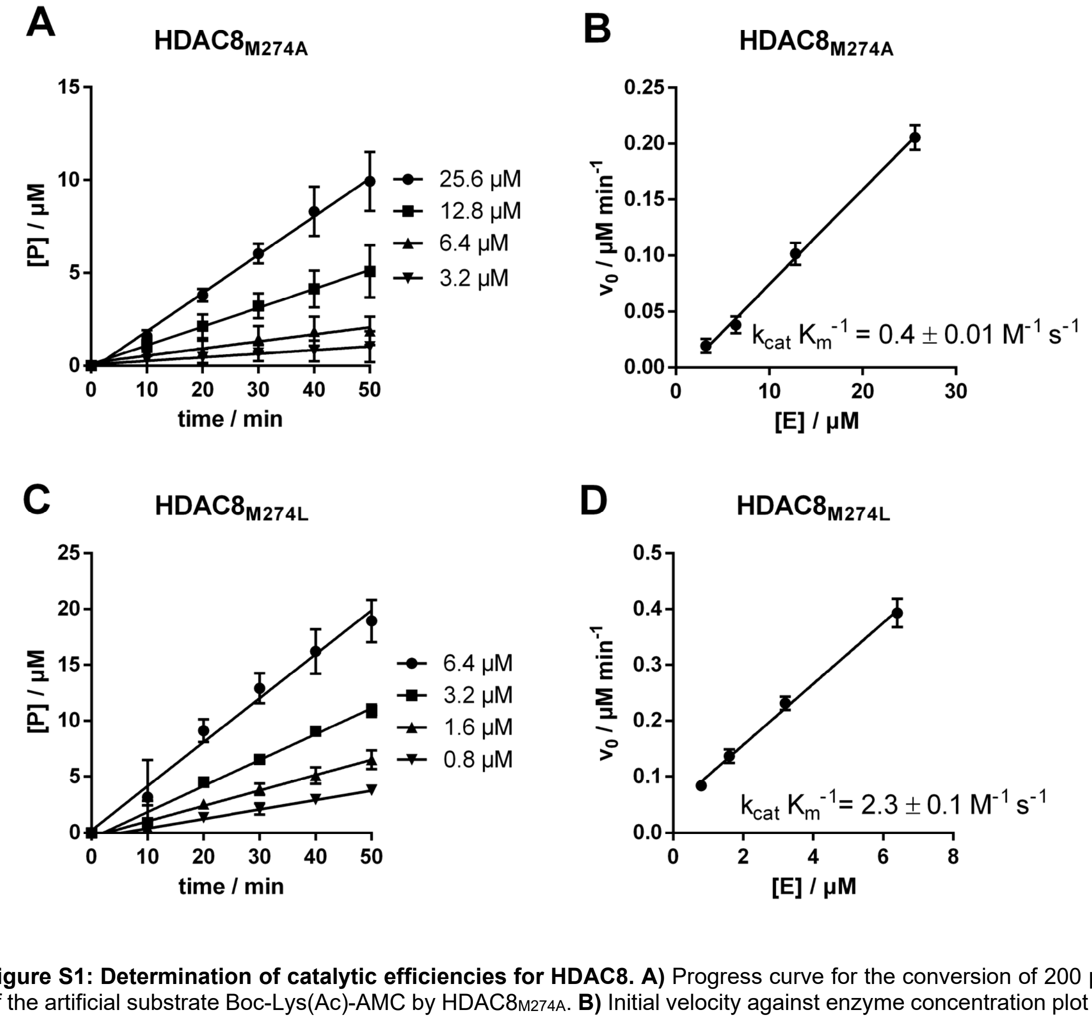

Figure S1: Determination of catalytic efficiencies for HDAC8. A) Progress curve for the conversion of 200 µM of the artificial substrate Boc-Lys(Ac)-AMC by $HDAC8_{M274A}$. **B)** Initial velocity against enzyme concentration plot for $HDAC8_{M274A}$. **C)** Progress curve for the conversion of 200 µM of the artificial substrate Boc-Lys(Ac)-AMC by $HDAC8_{M274L}$. **D)** Initial velocity against enzyme concentration plot for $HDAC8_{M274L}$.

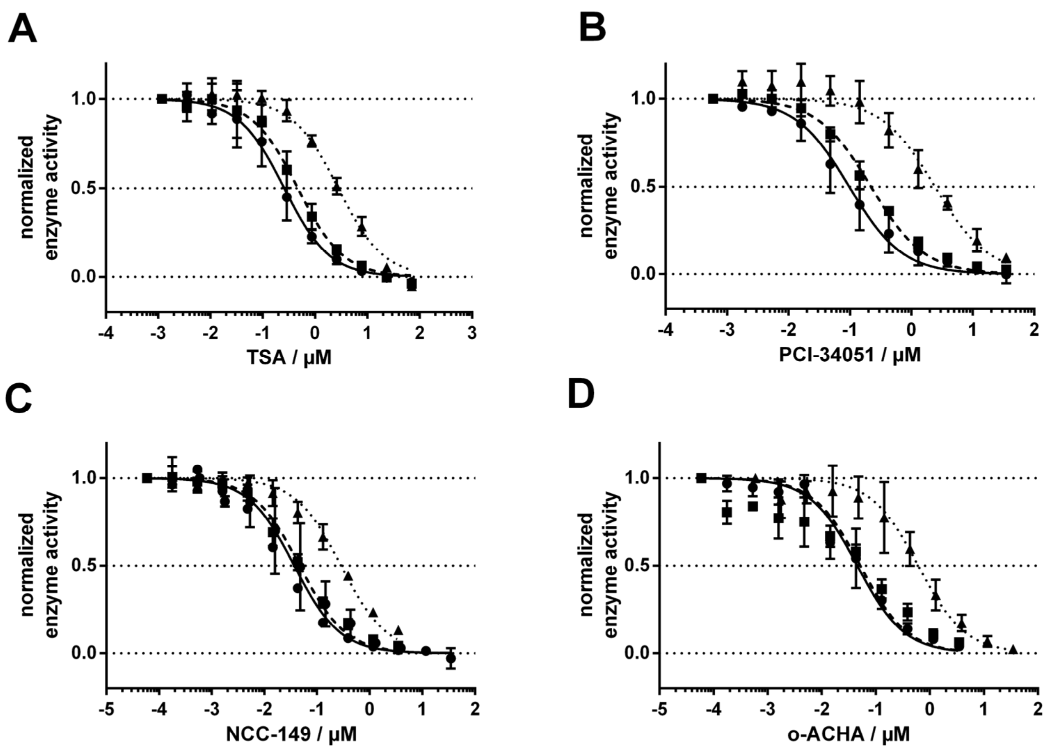

Figure S2: Dose-response curves for IC_{50} determination. A) Dose-response curve for the IC_{50} determination of TSA for $HDAC8_{wt}$ (solid line), $HDAC8_{M274L}$ (meshed line) and $HDAC8_{M274A}$ (dotted line). **B)** Dose-response curve for the IC_{50} determination of PCI-34051 for $HDAC8_{wt}$ (solid line), $HDAC8_{M274L}$ (meshed line) and $HDAC8_{M274A}$ (dotted line). **C)** Dose-response curve for the IC_{50} determination of NCC-149 for $HDAC8_{wt}$ (solid line), $HDAC8_{M274L}$ (meshed line) and $HDAC8_{M274A}$ (dotted line). **D)** Dose-response curve for the IC_{50} determination of o-ACHA for $HDAC8_{wt}$ (solid line), $HDAC8_{M274L}$ (meshed line) and $HDAC8_{M274A}$ (dotted line).

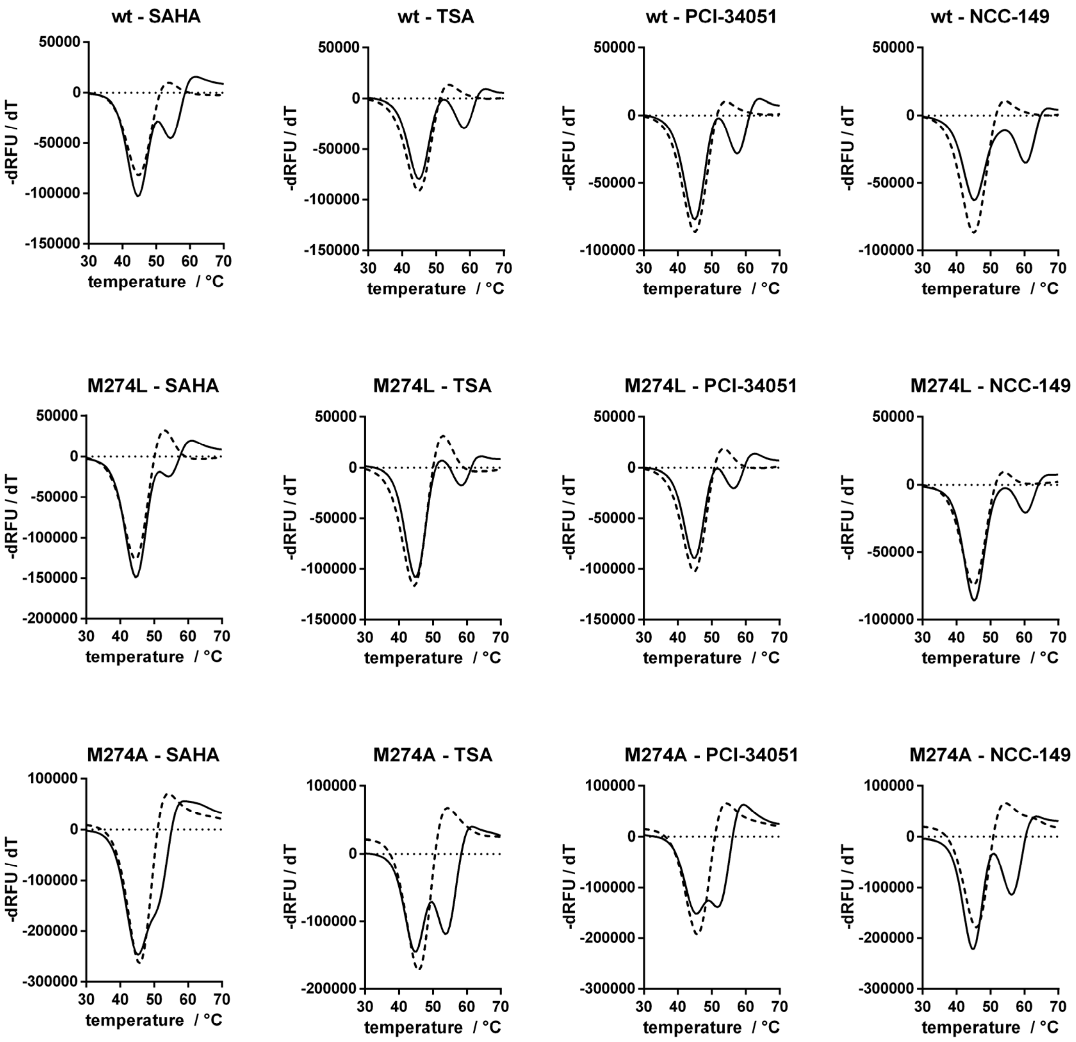

Figure S3: Overview of thermal shift assay for the binding of SAHA, TSA, PCI-34051 and NCC-149 on HDAC8$_{wt}$, HDAC8$_{M274A}$, and HDAC$_{M274L}$.

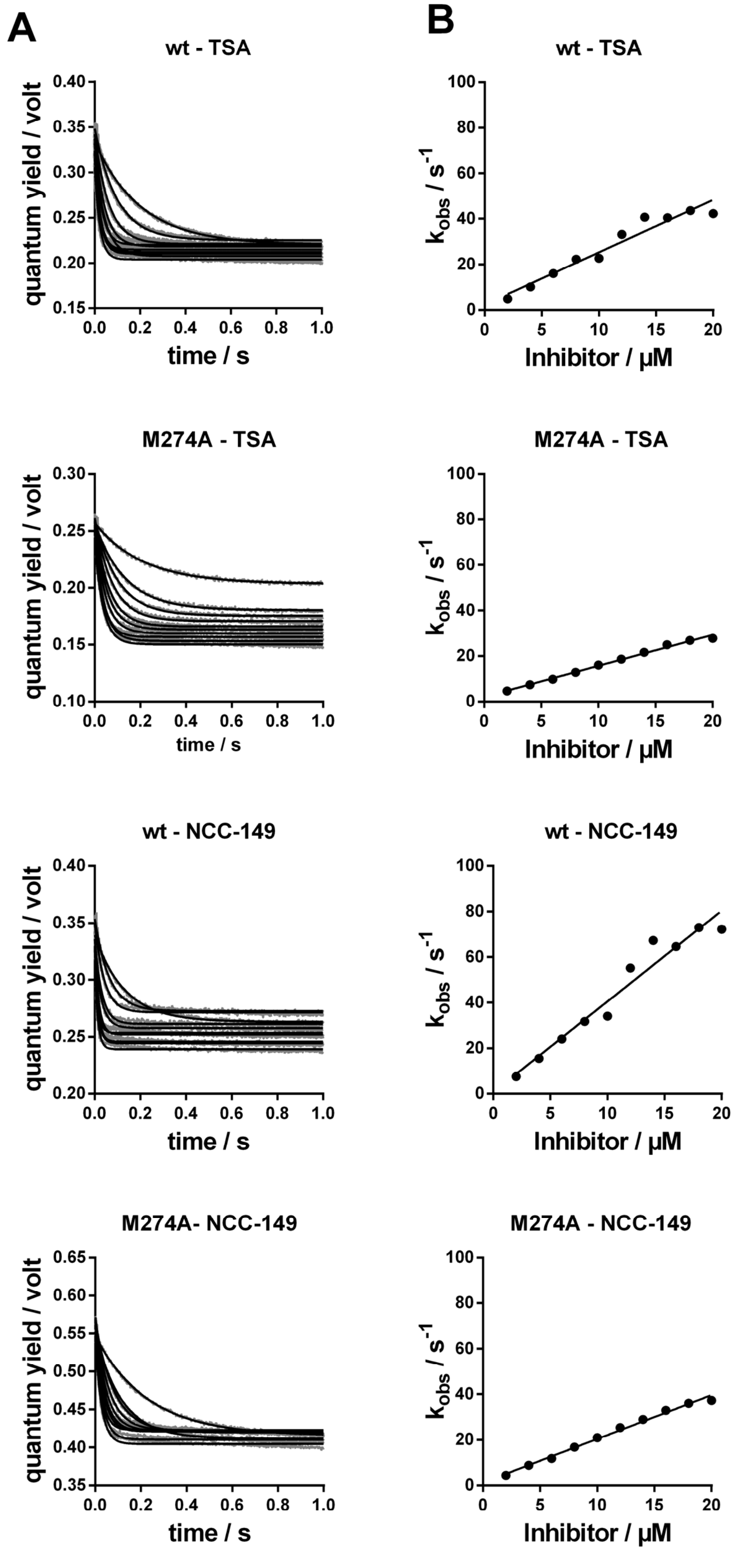

Figure S4: Determination of the rate constant of assotiation via stopped-flow. A) Time dependent loss in intrinsic tryptophane fluorescence upon binding of a inhibitor to HDAC8. Curves were fitted against a one-phase decay exponential using GraphPad Prism. **B)** k_{obs} against inhibitor concentration plot for the determination k_{on}.

A)

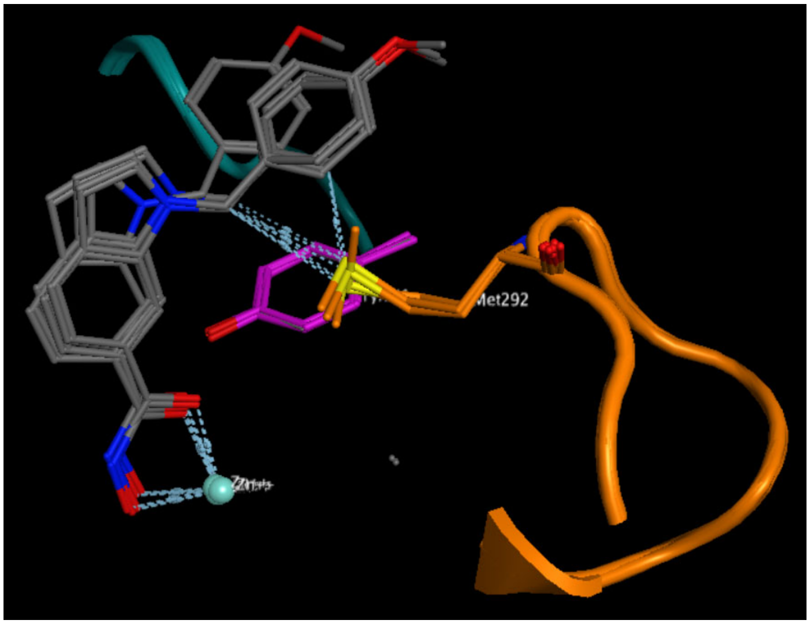

B)

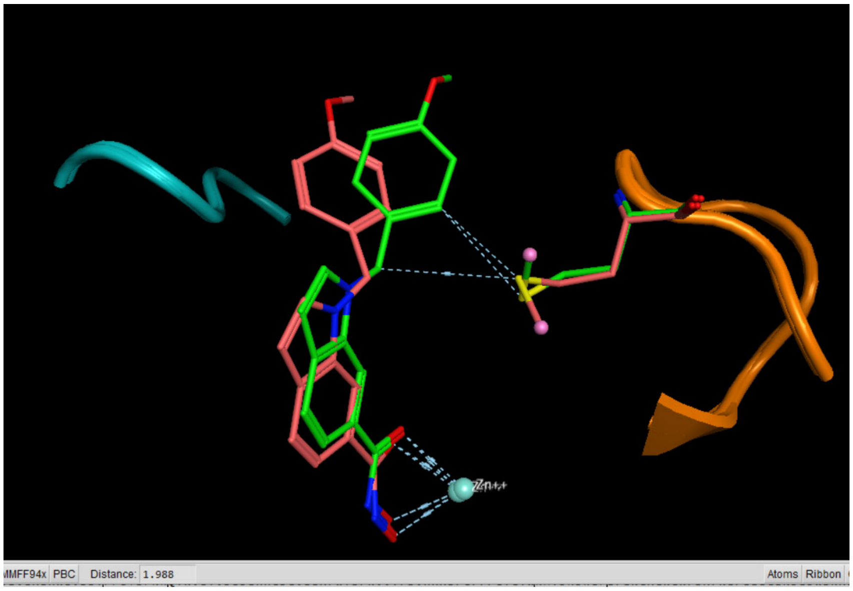

Figure S5: A) Overlay of HDAC8-monomers from the same crystal structure of tetrameric HDAC8 from *Schistosoma mansoni* (PDB-ID: 6HSF). The L1 loop (cyan) and L6 loop (orange) are shown as tubes and the catalytic zinc ions as cyan spheres. The HDAC8 selective pocket is defined by catalytic Y341 (magenta). Ligands and the methyl group of M292 in different monomers show different orientations indicating some conformational flexibility. The distance between the methyl carbon atoms of M292 is between 0.24 and 2.2 Å. **B)** There is 2.0 Å distance between the methyl carbons of M272 in chain A (green) and C (light red). The corresponding ligand is colored correspondingly. Notably, the out-conformation of methionine in chain A corresponds to a closer ligand (green) indicating that out-conformation of methionine does not interfere with binding of ligand.

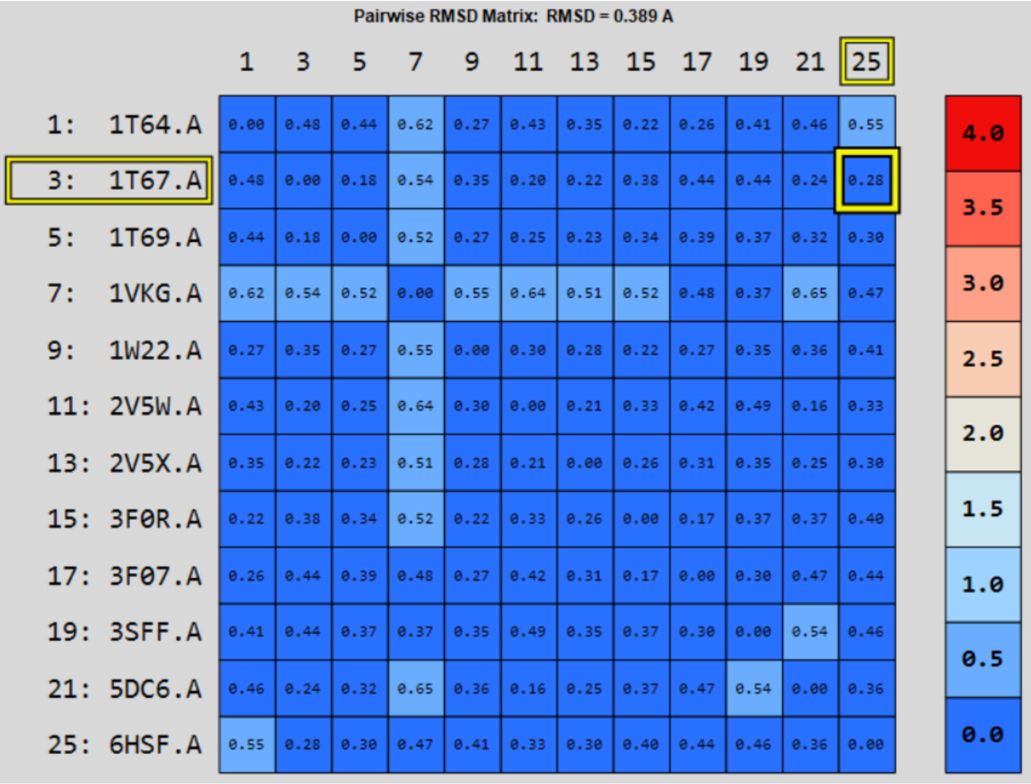

Pairwise RMSD Matrix: RMSD = 0.389 A

	1	3	5	7	9	11	13	15	17	19	21	25
1: 1T64.A	0.00	0.48	0.44	0.62	0.27	0.43	0.35	0.22	0.26	0.41	0.46	0.55
3: 1T67.A	0.48	0.00	0.18	0.54	0.35	0.20	0.22	0.38	0.44	0.44	0.24	0.28
5: 1T69.A	0.44	0.18	0.00	0.52	0.27	0.25	0.23	0.34	0.39	0.37	0.32	0.30
7: 1VKG.A	0.62	0.54	0.52	0.00	0.55	0.64	0.51	0.52	0.48	0.37	0.65	0.47
9: 1W22.A	0.27	0.35	0.27	0.55	0.00	0.30	0.28	0.22	0.27	0.35	0.36	0.41
11: 2V5W.A	0.43	0.20	0.25	0.64	0.30	0.00	0.21	0.33	0.42	0.49	0.16	0.33
13: 2V5X.A	0.35	0.22	0.23	0.51	0.28	0.21	0.00	0.26	0.31	0.35	0.25	0.30
15: 3F0R.A	0.22	0.38	0.34	0.52	0.22	0.33	0.26	0.00	0.17	0.37	0.37	0.40
17: 3F07.A	0.26	0.44	0.39	0.48	0.27	0.42	0.31	0.17	0.00	0.30	0.47	0.44
19: 3SFF.A	0.41	0.44	0.37	0.37	0.35	0.49	0.35	0.37	0.30	0.00	0.54	0.46
21: 5DC6.A	0.46	0.24	0.32	0.65	0.36	0.16	0.25	0.37	0.47	0.54	0.00	0.36
25: 6HSF.A	0.55	0.28	0.30	0.47	0.41	0.33	0.30	0.40	0.44	0.46	0.36	0.00

Figure S6: RMSD-analysis of superimposed crystal structures of 11 human HDAC8 (PDB-IDs 1T64, 1T67, 1T69, 1VGK, 1W22, 2V5W, 2V5X, 3F0R, 3F07, 3SFF, 5DC6) and smHDAC8$_{H292M}$ from *Schistosoma manson* (PDB-ID 6HSF). Alignment is based on 10 conserved active site amino acids (H142, H143, F152, D178, H180, F208, D267, P273, M274, Y306). The average pairwise RMSD value is 0.389 Å indicating that the positions of active site amino acids in HDAC8 are highly defined.

Table S1: Catalytic efficiencies and melting points for HDAC8$_{wt}$, HDAC8$_{M274L}$ and HDAC8$_{M274L}$.

	catalytic efficiency / $M^{-1} s^{-1}$	T_m / °C
HDAC8$_{wt}$	24 ± 3	45.11 ± 0.18
HDAC8$_{M274L}$	2.3 ± 0.1	44.95 ± 0.30
HDAC8$_{M274A}$	0.4 ± 0.01	45.57 ± 0.19

Table S2: IC_{50}, thermal shift and stopped-flow data for HDAC8$_{wt}$, HDAC8$_{M274L}$ and HDAC8$_{M274L}$.

	IC_{50} / µM				
	SAHA	TSA	PCI-34051	NCC-149	o-ACHA
HDAC8$_{wt}$	2.0	0.2	0.1	0.04	0.04
HDAC8$_{M274L}$	1.6	0.5	0.2	0.05	0.05
HDAC8$_{M274A}$	11.0	2.8	2.0	0.3	0.6
	T_m / °C				
HDAC8$_{wt}$	54.79 ± 0.33	58.77 ± 0.34	58.02 ± 0.18	60.82 ± 0.18	-
HDAC8$_{M274L}$	54.85 ± 0.38	58.77 ± 0.63	56.90 ± 0.37	60.73 ± 0.13	-
HDAC8$_{M274A}$	51.15 ± 0.41	54.47 ± 0.63	52.60 ± 0.25	56.90 ± 0.27	-
	k_{on} / s^{-1}				
HDAC8$_{wt}$	-	2.30 ± 0.20	-	3.90 ± 0.30	-
HDAC8$_{M274A}$	-	1.35 ± 0.03	-	1.92 ± 0.06	-

Table S3: Pairwise RMSD-values in Å between active sites in x-ray structures of smHDAC8 (PDB-ID: 6HSF) human HDAC8 structures with indicated PDB-Id's. The amino acid (AA) code of PDB-ID 1T69 is used.

	AA	1T64	1T67	1T69	1VKG	1W22	2V5W	2V5X	3F0R	3F07	3SFF	5DC6	Mean
**Catalytic**	H142	0.421	0.214	0.392	0.176	0.287	0.152	0.283	0.233	0.395	0.412	0.414	**0.31**
	H143	0.357	0.282	0.240	0.265	0.173	0.226	0.315	0.207	0.254	0.227	0.246	**0.25**
	Y306	0.199	0.365	0.305	0.654	0.274	0.246	0.350	0.277	0.434	0.584	0.265	**0.36**
**Zinc chelating**	D178	0.246	0.216	0.227	0.427	0.205	0.264	0.175	0.196	0.175	0.256	0.104	**0.23**
	D267	0.246	0.148	0.160	0.451	0.146	0.297	0.240	0.387	0.319	0.202	0.237	**0.26**
	H180	0.113	0.249	0.087	0.168	0.105	0.143	0.094	0.102	0.239	0.108	0.208	**0.15**
**Hydrophobic binding tunnel**	F152	0.862	0.425	0.423	0.687	0.723	0.759	0.463	0.570	0.377	0.237	0.798	**0.57**
	F208	0.504	0.287	0.287	0.160	0.178	0.219	0.167	0.290	0.264	0.289	0.281	**0.27**
**L6 loop**	P273	0.897	0.192	0.206	0.633	0.577	0.222	0.366	0.681	0.753	0.726	0.280	**0.50**
	M274	0.892	0.332	0.485	0.624	0.729	0.302	0.392	0.582	0.759	0.899	0.261	**0.57**

Table S4: Primers used for point mutations of HDAC8.

#name	5'-sequence-3'
M274L_for	GCGGGTGACCCGTTATGCAGCTTTAAC
M274L_rev	GTTAAAGCTGCATAACGGGTCACCCGC
M274A_for	GCGGGTGACCCGGCATGCAGCTTTAAC
M274A_rev	GTTAAAGCTGCATGCCGGGTCACCCGC
